# Patients' Contributions to Hospitals

**Published:** 1888-06-09

**Authors:** W. Burdett-Coutts


### Patients' Contributions to Hospitals.

By Mr. W. Burdett-Coutts, M.P.

*(Continued from p. 131.)*

### Guy's Hospital.

Guy's Hospital next claims our attention as having developed the Pay System to a considerable extent, twenty beds out of five hundred in the general wards being set aside for patients who can and will, either of themselves or through friends and employers, pay £1 1*s.* per week; but they have no privileges at all over the free patients. There is a private ward, with twelve separate compartments, for patients who pay £3 3*s.* a week. These, in addition to the private room, have superior furniture and surroundings, "and a more *recherché* diet." The secretary says: "Patients either pay weekly, or in a lump sum when they leave the hospital. We have no bad debts." These two sources produced last year the considerable sum of £1,874 6*s.* 4*d.* In addition to this there is a sum of £670 12*s.* from out-patients, at threepence per week, paid for their supply of medicine. In the three-guinea wards patients pay their medical attendants. It is contemplated to enlarge this department and appoint a resident medical officer to it, in which case no charge for medical attendance will be made, unless the patient desires to have his own medical attendant, or one of the physicians or surgeons of the hospital, who would of course charge their own fees. The secretary of Guy's is of opinion that it would be impracticable to have a graduated scale of payments, and implies that the only inducement held out to patients at this hospital to occupy the pay beds is a statement of the fact that they are charged for; but it is difficult to see why a patient should occupy a guinea bed when the same patient, without inquiry or restriction, can occupy a free bed alongside of it. The secretary thinks: "In a large hospital it would be an inquisitorial and thankless task to attempt to ascertain the means of patients—only those are admitted who are suffering acutely from disease or accident, while the majority are sent away as being ineligible on account of the less grave character of their complaints. If it were put to the applicants for admission that they must undergo an ordeal beforehand, similar to that enforced by the agents of the Charity Organisation Society, we might as well shut up shop altogether."

But on turning to his account of the Pay System in the Out-patients' Department I find that—"Since January, 1884, out-patients are required to pay threepence for their weekly supply of medicine. In cases of necessity and extreme poverty no payment is expected; but on the patient's appearing a second time he or she is required to produce *a paper, stamped by the Charity Organisation Society of the district* in which they live, certifying their inability to pay."

I am little disposed to favour the methods of the Charity Organisation Society, but I hardly understand why those methods should be more onerous to the in-patient of the hospital than to the out-patient. In return for £400 from the Hospital Saturday Fund, largely derived from workshops in South London, they give *carte blanche* to the promoters of the fund, which enables them to send any number of in and out patients to the hospital, provided they are physically suitable. If these letters find their way to the workshops from which the money came, which I have no doubt is the case, the Pay System is preserved, and indeed somewhat generously interpreted in this instance. The secretary states: "The cost of an occupied bed amounts to £71 per annum, so that the payments made by patients (£54 10*s.*) do not cover their expenses." Of course not; it never is contemplated in the Pay System that they should do so; and he comes to the kernel of the question when he adds: "On the other hand, the arrangement enables the governors to keep a large number of beds open for the reception of free patients." In this case the twenty beds supply fifteen more beds. Multiply this result, derived from a very limited application of the principle, and you will understand what the pay system would do for our hospitals. Finally, the Secretary remarks: "We are very well satisfied with the experiment of requiring people to pay something, however small, for the benefits they receive; but unless other establishments adopt the same principle, the moral effect on the population generally will be very slight."

### Pay System must be Universal to be Effective.

It is obvious that the universality of the system—by which, I must repeat, I do not mean that every one should pay, but that every hospital should take payment, or take none but poor patients—is necessary not only to its moral effect, but to its practical success.

If, on the one hand, there is no inducement within the hospital, such as that to which I have alluded above, to a patient to pay for his treatment; nor, on the other, from without, any implied obligation upon him or inquiry into his capacity to do so, it is certain that he will prefer to be treated for nothing. Some may say, "We prefer the universality of the free system." But have you got it, or can you even reach it? Take the cases of the parish infirmaries, which admit and treat at the expense of the rates great numbers of absolutely poor patients. How do the latter differ from the corresponding class who are admitted free to hospitals? Is there any real or fair test by which you separate off a portion of the absolutely poor and admit them to free treatment in London hospitals, and send another portion into the parish infirmary? I am not speaking of the aged or incurable sick, but of the large number of absolutely poor patients in parish infirmaries, who are in exactly the same condition as the absolutely poor admitted to the hospitals. We all know which institution they prefer. Why should some be favoured and others rejected? This raises a question, upon which I would refer you to the account already given of the hospital at Christiania, but which is far too large to be included in this paper, viz., that of State aid to hospitals. But the fact which I have pointed out is most pertinent to the subject of this paper, and appears to me to painfully illustrate the distorted and illogical basis upon which our present system rests.

### King's and University College Hospitals.

At King's College Hospital it is stated paying patients are taken into the ordinary wards at a charge of one guinea a week, but as the receipts from this source indicate only one occupied bed throughout the year, the patients evidently prefer the free system, which is certainly not to be wondered at.

At University College Hospital the "People's Contribution Fund" appears to be based upon proper Pay System principles, in that it is provided that (1) "representative members of the working classes would become annual or life governors, and participate in the management of the hospital"; and (2) "all subscribers would have the advantage of finding a supply of hospital tickets in the possession of neighbours or fellow-workmen, their own representatives." These provisions resemble the practice so thoroughly carried out in Sunderland; but the limited success of the experiments illustrates the contrast I have already pointed out between the conditions of the Sunderland and London populations. I find that the employés of a number of firms, etc., in the neighbourhood contribute. The annual subscriptions are £122, and the donations £138, in return for which I presume the above privileges are given, and to that extent this fund appears to me to be well conceived. But it is evident that the most important part of the fund is charity pure and simple, for, of a total of £655 received in 1887, £394 came from the collecting-boxes placed in hotels, shops, public-houses, etc. I do not know how far this part of the scheme involves the privileges of free letters or life governorships to the place where the box stands, but it would seem difficult to secure that the contributors to the boxes, either individually or in different classes, should get a return for their money.

Metropolitan Hospital, Kingsland Road.

An important experiment is being tried at the Metropolitan Hospital, Kingsland Road, but I regret it has not been sufficiently long in existence to supply important material to this paper. As I am anxious that any suggestions or arguments that I may use should be based upon ascertained facts, it is worth while referring to the Out-Patient Provident Department at the Royal Albert Hospital, Devonport, as bearing upon this. The Metropolitan Hospital admits paying patients into both the in and out patient departments. The hospital is on provident principles throughout. The subscription is fourpence a-month for adults, and twopence a-month for children; sixpence a-month includes all children in a family under the age of sixteen. One penny is charged for each prescription made up, beyond which there is no charge for medicine. There are other interesting details, but the gist of the experiment as connected with the subject of this paper lies in the fact that no one is admitted free, "except accidents and cases of urgency"; that "no single man earning more than twenty-five shillings per week, or man, wife and family earning forty shillings, can join the Provident Department"; that inquiries will be made if it is thought an applicant is able to pay the regular fees of a medical man." These regulations appear to be true to the Pay System. It is spoken of as the "Out-Patient Provident Department," but I notice that "when necessary persons will be admitted as in-patients." The plan appears to have been cordially taken up by working people of the neighbourhood. It was started on November 30th last; there are already 1,800 members' books issued, "representing 4,500 lives." The secretary states that it has been "a distinct success"; and adds that the system "assists in teaching the poor to be provident; assists in preventing improper objects from obtaining hospital relief; and increases the resources of the charity." These are so nearly the objects of an adoption of the Pay System that I shall watch the progress of this hospital with the greatest possible interest.

German Hospital, Dalston.

The German Hospital, Dalston, in addition to its 125 general beds, has six private rooms, taking patients who speak the German language for £1 11*s.* 6*d.* and £2 2*s.* per week, which the Secretary states pays very well, as the cost of an in-patient in the wards amounts to £1 3*s.* 6*d.*, and the average received from the above paying patients last year was £1 16*s.* 2*d.* per head per week. The Committee sometim s make exceptions and forego the charge, of which there were six instances out of thirty-nine cases last year. These private wards are considered a great boon.

At the Temperance Hospital, with sixty-six beds, which I treat with the general hospitals of London, paying patients are taken to both departments and to both general and private wards; in-patients, on the recommendation of someone known to the authorities of the hospital, and who is acquainted with the patient's means; out-patients with a subscriber's letter being admitted free, but without such letter paying one shilling. There are very few in-patients and about forty paying out-patients per week. The total receipts from the two sources were £260 for the year.

These are the only instances of the Pay System among the general hospitals in London, and even in them, as will be obvious, the system is so partial and is attended with so many difficulties when confined to isolated instances, that it affords no fair basis for a calculation of its financial effects if generally practised.

(*To be continued*).

